# Effect of Molar Substitution on the Properties of γ-Hydroxypropyl Starch

**DOI:** 10.3390/molecules27072119

**Published:** 2022-03-25

**Authors:** Xue-Li Liu, Yi-Fan Chen, Jing-Jing Yang, Si-Jin Li, Hua-Le Xie, Tian-Lin Ma

**Affiliations:** 1 College of Material and Chemical Engineering, Chuzhou University, Chuzhou 239012, China; c1206108643@163.com (Y.-F.C.); yjj122019@163.com (J.-J.Y.); 13515685591@163.com (S.-J.L.); mw18355941713@163.com (H.-L.X.); 2 School of Chemistry & Chemical Engineering, Anhui University, Hefei 230601, China

**Keywords:** γ-hydroxypropyl starch, molar substitution, physicochemical properties

## Abstract

A new type of hydroxyalkyl starch, γ-hydroxypropyl starch (γ-HPS), was prepared by etherification of alkali-activated starch with 3-chloropropanol. The reaction efficiency, morphological change, thermodynamic and apparent viscosity properties, and other physicochemical characteristics were described. The molar substitution (MS) of modified whole starch was determined to be 0.008, 0.017, 0.053, 0.106, and 0.178, with a ratio of 5%, 15%, 25%, 35%, and 45% 3-chloropropanol to starch (*v*/*w*), respectively. Compared to native starch, the granular size and shape and the X-ray diffraction pattern of γ-HPS are not very different. For low-substituted γ-HPS, the implications may be less evident. Thermal stability measurements by means of thermogravimetric analyses and differential scanning calorimetry (TGA-DSC) proved that thermal stability was reduced and water retaining capacity was increased after hydroxypropylation. Furthermore, the findings also showed that the solubility, light transmittance, and retrogradation of γ-HPS pastes could be improved by etherification. The greater the MS of the γ-HPS, the more its freeze–thaw stability and acid resistivity increased. In this study, we provide relevant information for the application of γ-HPS in food and non-food industries.

## 1. Introduction

As an important polysaccharide, starch has received considerable attention in recent years [1,2]. However, native starch is always modified with chemical, physical, or biological treatments [3,4,5] due to its non-negligible disadvantages, such as low gelatinization temperature, insolubility in cold water, weak anti-retrogradation ability, heat variable viscosity, easy swelling, etc. [6] Etherification is one of the most common modification methods. Among etherified starches, hydroxyalkyl starch has many advantages, such as better pH stability [7], good liquidity and solubility [8], good swelling capacity [9], high dispersion and adhesion [10], etc., giving it a place in various industries. Moreover, hydroxypropylation is a chemical modification method commonly used on starch, achieved using 1,2-epoxypropane as the etherifying reagent [11]. Classical hydroxypropyl starch is obtained by introducing the 2-hydroxypropyl group onto the O-2, O-3, and O-6 of a glucosyl unit. Currently, some reports about classical hydroxypropyl starch synthesis have been presented [12,13,14]. We previously developed a new type of hydroxypropyl starch, namely γ-hydroxypropyl starch (γ-HPS), i.e., with the hydroxyl group on the C-3 position of the propyl group [15], and two simple and efficient methodologies for determining molar substitution (MS). It is well-known that structure determines nature, whereas utility is determined by nature. The properties of modified starch are affected by the degree of modification [16,17]. Therefore, the objective of this study was to investigate the physicochemical properties of γ-HPS, including its reaction efficiency, morphological change, thermodynamic and apparent viscosity properties, and other physicochemical characteristics, with all levels of MS ranging from 0.01 to 0.2. We studied the effectiveness of the chemical modification of native starch through γ-hydroxypropylation, in order to observe any improvements in its functional characteristics that might enhance its potential application in the food industry.

## 2. Materials and Methods

### 2.1. Materials

Corn starch, NaOH, alcohol, isopropanol, and 3-chloropropanol were purchased from Aladdin Reagent Co., Ltd., (Shanghai, China). All other reagents used in this work were of analytical grade.

### 2.2. Preparation of γ-HPS

Hydoxypropylated starch, with varying MSs, was prepared by producing a reaction between native corn starch and 3-chloropropanol, according to the procedure of Han [18] and Liu [15] with slight modifications. A sample (10 g, dry basis) and a solution of NaOH (0.6 g) in 95% isopropanol (100 mL) were added into a 250 mL bottle. The mixtures were stirred for 1 h at room temperature. The reaction was maintained at 45 °C for 12 h after 3-chloropropanol (0.5, 1.5, 2.5, 3.5, and 4.5 mL) was added. After the vacuum filtration step, the product was neutralized with dilute HCl (0.1 M), washed with a 95% aqueous ethanol solution three times, and then dried in an oven at 50 °C until the moisture content was reduced to 11–13%. The hydroxypropyl content of γ-HPS was determined according to the Zeisel-gas chromatographic method reported by Liu [15] and is expressed as a MS.

### 2.3. Determination of MS

MS was determined using Equation (1), as described by Liu [15], and the reaction efficiency was calculated using the ratio of experimental MS to theoretical MS. The values 162.14 and 58.08 in the following equations represent the molecular weight of AGU and C_3_H_6_O; W_P_ is the equivalent propyl oxide amount in 100 mg starch.

(1) $$\mathrm{MS}=\frac{W_{P}}{100-W_{P}}\times \frac{162.14}{58.08}\;$$

(2) $$\mathrm{Reaction}\;\mathrm{efficiency}\left(\%\right)=\frac{\mathrm{experimental}\;\mathrm{MS}}{\mathrm{theoretical}\;\mathrm{MS}}\times 100$$

### 2.4. Wide-Angle X-ray Diffractometry

The X-ray diffraction patterns of native corn starch (NCS) and γ-hydroxypropyl starch were tested with a Bruker X-ray diffractometer (D8 Advance, Bruker Corp., Middlesex, MA, USA) and a CuKα radiation detector (Bruker Corp., Middlesex, MA, USA) under the following conditions: 40 kV, 30 mA, and 1.5 s time counts. The diffractograms were registered at the Bragg angle (2θ) = 20°–80°.

### 2.5. Scanning Electron Microscopy

The granule morphologies of NCS and γ-HPS were observed with a JSM-6510LV ultra-scanning electron microscope (JEOL Ltd., Tokyo, Japan), following the reported literature [19].

### 2.6. Thermal Characterization

The thermal stability of the native and etherified starches was measured using an SDT-Q600. Thermogravimetric analysis (TGA) and differential scanning calorimetry (DSC) were combined. The sample (10 mg) was heated from 25 to 600 °C at a rate of 10 °C/min under the protection of ultra-pure nitrogen. The thermogravimetric curve and differential scanning calorimetry (DSC) curve were drawn by a computer.

### 2.7. Transmittance

The paste clarities of NCS and γ-HPS were determined according to the reported literature [20,21]. The sample (50 mg) was mixed with 5 mL of distilled water in a 10 mL graduated test tube with stopper, heated in a boiling water bath for 30 min, and then cooled to room temperature. The transmittance of the sample was measured at a wavelength of 620 nm with a spectrophotometer (Varian Cary 100, Varian Corp., Palo Alto, CA, USA); the distilled water was used as a blank. All analyses were carried out in triplicate, unless otherwise stated (the same applies below).

### 2.8. Starch Solubility

The solubility of native and modified starch was measured by using 50 mL of the corresponding starch emulsion solution (2%, *w*/*v*), which was heated in a boiling water bath for 30 min, then centrifuged (3000 r/min) for 20 min. The supernatant was dried and evaporated in a vacuum oven at 105 °C for 12 h. The solubility is expressed as a percentage, which we determined using the weight of starch contained within the supernatant and the total weight of the sample as follows:

(3) $$\mathrm{Solubility}\left(\%\right)=\frac{\mathrm{Weight}\;\mathrm{of}\;\mathrm{water}\;\mathrm{soluble}\;\mathrm{starch}\left(g\right)\times 100}{\mathrm{Total}\;\mathrm{weight}\;\mathrm{of}\;\mathrm{sample}\left(g\right)}$$

### 2.9. Retrogradation

To analyze retrogradation, we first measured 100 mL of aqueous suspension of starch (1%, *w*/*v*). Subsequently, we heated it in a boiling water bath under constant agitation for 20 min, and then cooled it down to room temperature. We poured the solution into a 100 mL measuring cylinder, diluted it with laboratory-pure water to another volume, mixed it, and let it stand for a certain amount of time. We recorded the volume of the supernatant every 12 h. Retrogradation was determined by the percentage of volume of supernatant over time.

### 2.10. Freeze–Thaw Stability

The freeze–thaw stability of NCS and γ-HPS was measured by following the method outlined in the literature [22], with a few small changes. The starch suspension (2%, *w*/*v*) was heated, in order to gelatinize, in a boiling water bath under constant agitation for 20 min, using a beaker with a scale. It was then cooled down and diluted to another volume with laboratory-pure water. Finally, it was poured into a centrifuge tube, covered with a lid, and put in a fridge. An alternating freeze–thaw cycle was conducted, freezing for 20 h at −18 °C and thawing for 4 h at 25 °C. The centrifuged water was separated and measured as a baseline for freeze–thaw stability.

(4) $$\mathrm{Syneresis}\left(\%\right)=\frac{\mathrm{Water}\;\mathrm{separated}\left(g\right)\times 100}{\mathrm{Total}\;\mathrm{weight}\;\mathrm{of}\;\mathrm{sample}\left(g\right)}$$

### 2.11. Apparent Viscosity

The apparent viscosity of the cooked starch and γ-HPS samples was determined using a rotational viscometer (NDJ-1, Lichen Instrument Tech. Co. LTD., Shanghai, China). The right amount of well-gelatinized starch solution (2%, *w*/*v*) was transferred to a proper beaker. The viscometer measured the viscosity change of an aqueous suspension sample with different MSs and pHs. We measured three times and calculated the mean value.

## 3. Results and Discussion

### 3.1. MS and Reaction Efficiency

**Figure 1:**
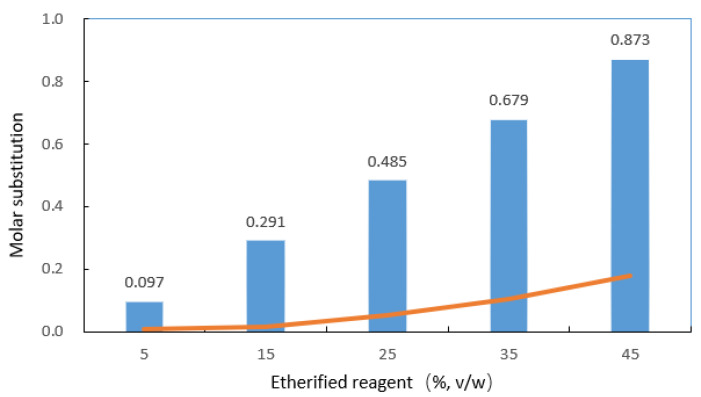
Effect of etherified reagent on molar substitution of hydroxypropylated starches. (Histogram is theoretical MS, trendline is experimental MS).

MS increased as the input quantity of etherified reagent increased. The MSs obtained for the γ-HPS were 0.008, 0.017, 0.053, 0.106, and 0.178 for 5%, 15%, 25%, 35%, and 45% 3-chloropropanol to starch (*v*/*w*), respectively (Figure 1). This observation is in line with the hydroxypropylation process of canna and maize [23], pigeon pea [24], and white yam [25] starches. The FDA stipulates that all hydroxypropylated starches must not surpass the maximum permissible level in food applications, that is to say, the MS cannot be more than 0.2 [26]. As shown in Figure 1, the theoretical value is much higher than the experimental value. The reaction efficiency for the preparation of γ-HPS was between 6% and 21%, depending on the concentration of 3-chloropropanol. The reaction efficiency was influenced by many factors. Altering reaction conditions (pH, temperature, reaction time, swelling-inhibiting salt type and concentration, etherified reagent type, molar ratio, etc.) impacted both MS levels and the uniformity of reaction within granules [27,28]. However, optimizing the proportion parameters and forecasting the optimal process conditions were not the focus of this study.

### 3.2. Wide-Angle X-ray Diffraction Pattern

**Figure 2:**
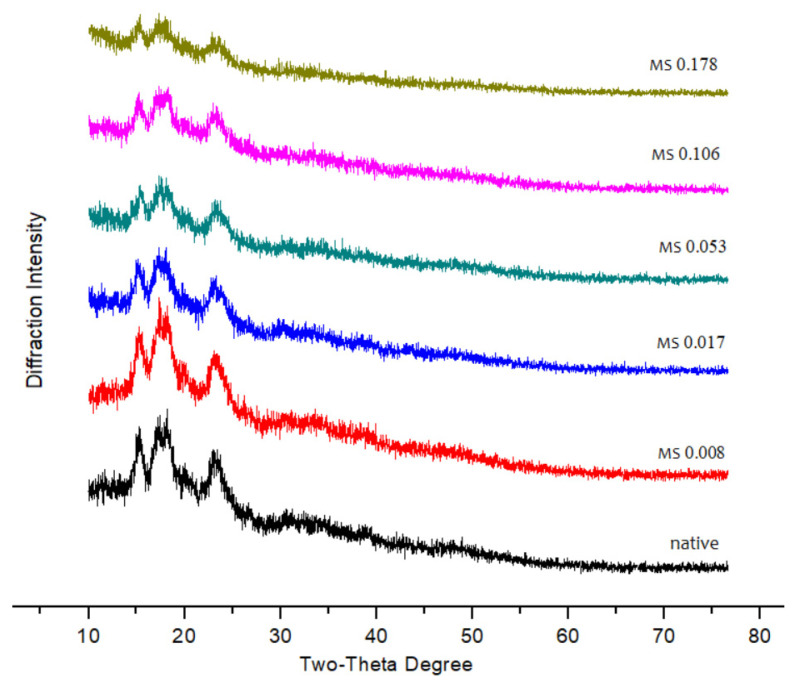
X-ray diffraction patterns of native and hydroxypropylated starches.

As exhibited in Figure 2, the X-ray diffractograms of NCS and γ-HPS meet the “A” pattern characteristic of cereal starches. There is a prominent peak at 15°, a doublet at 17° and 18°, and only one peak at 23°. Similar patterns were also observed after hydroxypropylation. Other similar observations were reported for plantain [26], rice [29], and hydroxypropylated starches with similar peaks at 2θ = 15, 17, and 23. In this study, when increasing the extent of etherification, the strength of the doublet peaked around 2θ = 17°, while 18° weakened. Meanwhile, a slight weakening in the intensity of the peaks at 2θ = 15° and 23° was observed. The initial results indicate that the crystalline region of the starch may undergo changes after the etherification reaction [30,31]. This is similar to the findings of reports on pigeon pea [24] and white yam starch [25]. In contrast, a slight increase in corresponding X-ray intensity was found for hydroxypropyl canna starch [23]. The explanation for this phenomenon may be the low level of hydroxypropyl modification used in this study.

### 3.3. Morphology of γ-HPS

**Figure 3:**
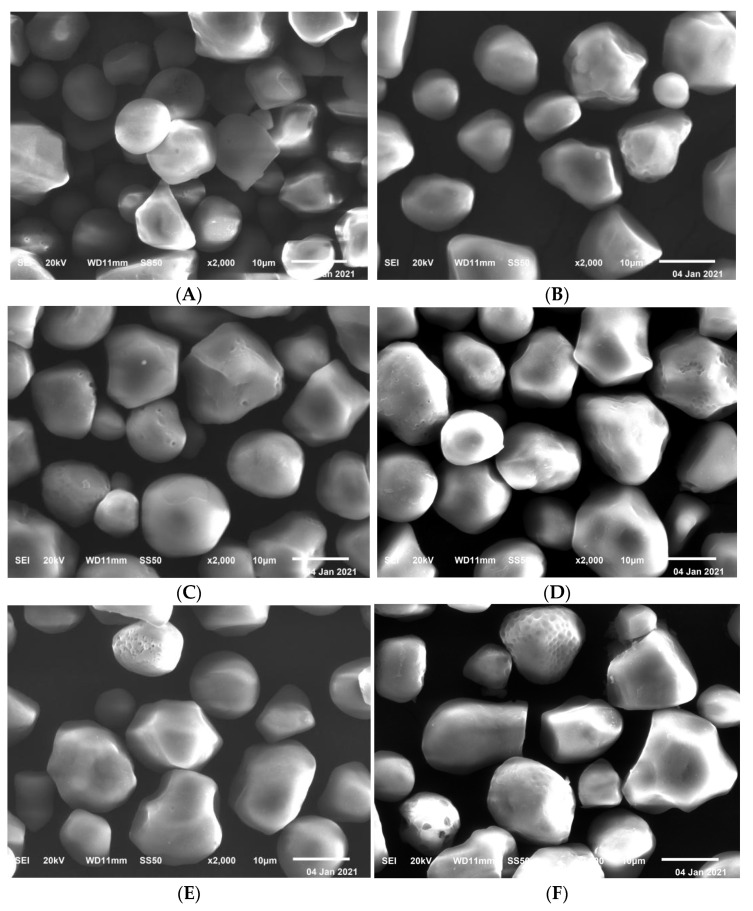
Scanning electron micrographs (2000×, bar as 10 μm) of native starch (**A**) and γ-HPS at MS = 0.008 (**B**), MS = 0.017 (**C**), MS = 0.053 (**D**), MS = 0.106 (**E**), and MS = 0.178 (**F**).

The SEMs of the NCS and γ-HPS are demonstrated in Figure 3. The granules of NCS were almost rounded or oval or disk-shaped, with a slick surface. In our study, hydroxypropyl modification at all levels of substitution did not completely change the form or surface features of the granules [23]. Furthermore, after hydroxypropylation, the granules stayed intact, as no breakage occurred. Treatment of the NCS granules with 3-chloropropanol resulted in changes on the granule’s surface. The surface corrosion consisted of bumps and hollows, comparable to the moonscape. Compared with the small size granules, the large size granules were more likely to be affected. This is similar in the hydroxypropylation of pigeon pea starch [24]. The reason for this may lie in the differences in structure and fragility of the granules. The above observations could be attributed to native starch morphology and the preparation process of the hydroxypropyl starch derivatives.

### 3.4. Thermal Properties

In this study, thermogravimetric analysis (TGA) and differential scanning calorimetry (DSC) were combined to investigate the thermal stability of NCS and γ-HPS. The curves of TGA experiments for the whole series of starch derivatives are presented in Figure S1. As we can see in the above curves, native and modified starches exhibit at least three decomposition stages. For example, in the case of native starch, below 100 °C, there is a small weight loss in the curve graph. Generally, this sort of situation is caused by a loss of adsorbed and bound water [32]. Between 260 and 340 °C, there is significant weight loss, which occurs in the second stage. The prime reason for this may be the depolymerization and degradation of products, such as carbon dioxide, carbon monoxide, water, acetaldehyde, and furan, in a non-oxidation process. Above 340 °C, this trend in weight loss is relatively modest. The last decomposition stage corresponds to carbonization and the total degradation of intermediate products at high temperatures. Similar results are seen in the TGA-DSC curves of γ-HPS, except for the narrow magnitudes. According to the TGA curve of γ-HPS, the initial and final temperatures of the thermal decomposition reaction decreased as MS increased, which can be observed at the following temperature ranges: 220–320 °C (MS 0.008), 210–320 °C (MS 0.017), 200–320 °C (MS 0.053), 200–320 °C (MS 0.106), and 200–310 °C (MS 0.178). Meanwhile, the second decomposition stage shows an endothermic peak at 319.16 °C, which corresponds to the fusion of the native starch [33]. The temperature of the endothermic peak decreased with the increase in MS, such as at 266.14 °C (MS 0.008), 264.3 °C (MS 0.017), 255.66 °C (MS 0.053), 255.21 °C (MS 0.106), and 252.04 °C (MS 0.178). Therefore, the thermal stability of the original starch is reduced after hydroxypropylation. Conversely, we found that a separate endothermic peak from the first evaporation stage increased as MS increased. The temperature of the endothermic peak increased from 75 °C to 125 °C, which indicates a stronger water retaining capacity at higher MSs.

### 3.5. Paste Clarity

The light transmittance of native starch and γ-HPS was investigated. The results indicated that hydroxypropylation can improve paste clarity. Furthermore, the higher MS of hydroxypropyl starches can increase paste clarity. All of them are listed below: 2.3% (native), 7.6% (MS 0.008), 18.7% (MS 0.017), 30.3% (MS 0.053), 56.4% (MS 0.106), and 67.8% (MS 0.178). These results are in line with hydroxypropyl potato, corn, and amaranth starch [22,34]. Thus, the introduction of γ-hydroxypropyl substituents (-CH_2_CH_2_CH_2_-OH) should increase steric hindrance, prevent the accumulation and crystallization of amyloid chains, and effectively weaken the strength of inter-chain hydrogen bonds.

### 3.6. Solubility

**Figure 4:**
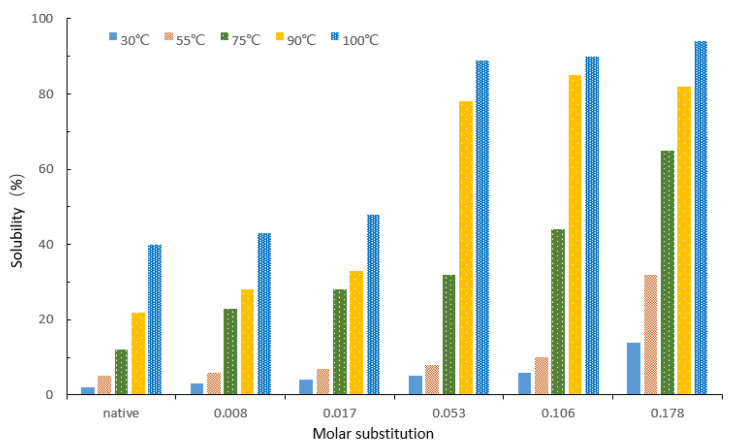
Solubility of native starch and γ-HPS at different temperatures and ratios of MS.

The solubility of NCS and γ-HPS was affected by factors such as temperature and modification extent (Figure 4). Just as with paste clarity, solubility is impacted by the introduction of γ-hydroxypropyl substituents (-CH_2_CH_2_CH_2_-OH). Solubility is proportional to the increased MS of γ-HPS. In the present study, γ-HPS with an MS of about 0.178 was not particularly soluble in normal temperature water. Meanwhile, hydroxypropyl starch showed a significant increase in solubility above 75 °C. Temperature is also a favorable factor. High temperatures resulted in an increase in solubility. The gelatinization temperature and higher MSs are crucial for improving solubility. Similar observations for hydroxypropylated sago starch [35] were reported.

### 3.7. Freeze–Thaw Stability

**Figure 5:**
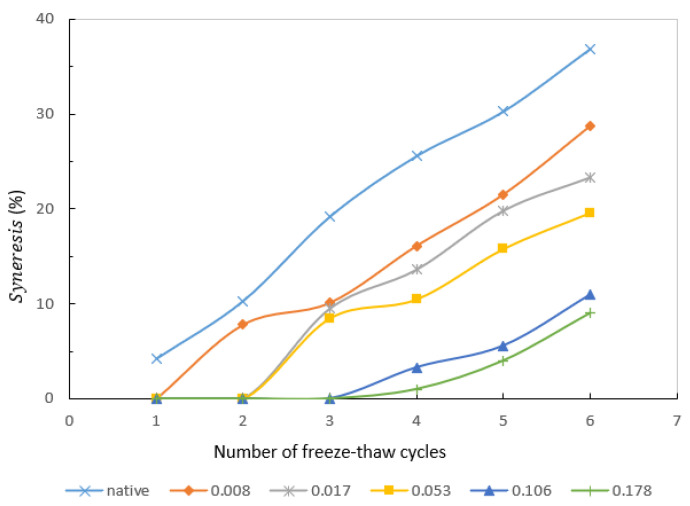
Syneresis (%) during freeze–thaw cycles for native and γ-HPS pastes.

There is an apparent difference in Figure 5 between NCS and γ-HPS. For native starch, the gel turned into a sponge-like material after only one freeze–thaw cycle. The freeze–thaw stability of modified starch gels improved dramatically after hydroxypropylation. Compared with native starch, the period yielding separated water was improved and exhibited better freeze–thaw stability (depending on the MS). In the second cycle, the hydroxypropylated starch of lower MS (0.008) began to precipitate water. For the starches with MS > 0.178, no syneresis was recorded until the fourth cycle. The more exposed the 3-hydroxypropyl group into starch chains, the better the effect of syneresis reduction. This phenomenon was also reported for sago starch [36].

### 3.8. Retrogradation

Table 1 shows the stability of NCS and γ-HPS put through a continuous record over 72 h. Native starch began to retrograde after 6 h and grow over time. Hydroxypropylation could effectively mitigate the syneresis in starch gels for 48 h (MS 0.178). Even in the lower substituted hydroxypropylated starch (MS = 0.02), the effect of anti-retrogradation was obvious. In addition, the boundary between water and gel grew more blurred. A reasonable explanation is that the interaction and structural arrangements between starch chains already were affected by the grafted γ-hydroxypropyl substituents (-CH_2_CH_2_CH_2_-OH), all of which can directly affect starch retrogradation.

### 3.9. Apparent Viscosity and Acid Resistivity

**Figure 6:**
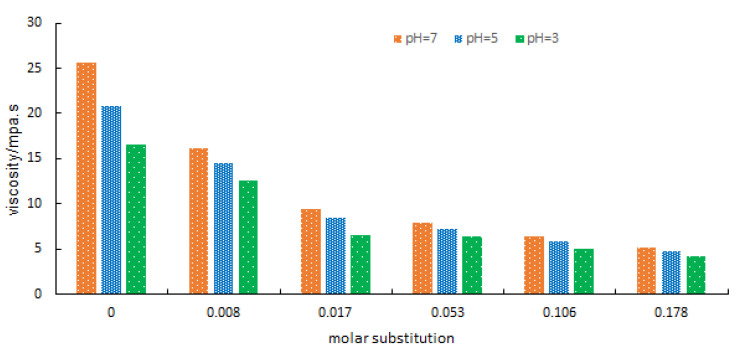
Effect of MS and pH on peak viscosity of native and γ-HPS.

The apparent viscosity of native and γ-HPS was measured at different concentrations and pH levels (Figure 6). The decrease in the viscosity of the γ-HPS, relative to that of MS, occurred due to introduction of the solubilizing hydroxypropyl group. Viscosity decreased when solubility increased. In addition, as pH decreased, the apparent viscosity of native and γ-HPS decreased. Nevertheless, the extent of the reduction in apparent viscosity decreased as MS increased, which indicated that hydroxypropylation may effectively increase acid resistance.

## 4. Conclusions

In this study, γ-HPS was successfully prepared with different levels of MS, ranging from 0.01 to 0.2, and the influence of MS on γ-HPS physicochemical properties was examined. After γ-hydroxypropylation, the functional parameters underwent significant changes, particularly the solubility and paste clarity, as well as the freeze–thaw and retrogradation stability. Some of these relevant functional parameters indicate what is needed to make starch derivatives useful in various industries. The low-substituted etherified γ-HPSs were tailored within the confines of the limits allowed by the appropriate regulation agencies regarding food application. Generally, the physicochemical characteristics enhance as the level of modification increases. Apart from the food sector, starch derivatives may also be relevant in other applications such as hydrogels and composite coating. Further investigations into the effectiveness of such applications of γ-HPS with a high degree of substitution are currently underway in our laboratory.

## Figures and Tables

**Table 1:** Retrogradation of native starch and γ-HPS.

Time/Sample ^1^	Native	A	B	C	D	E
6 h	3.6%	-	-	-	-	-
12 h	12.5%	2.2%	1.3%	-	-	-
24 h	25.2%	9.8%	5.1%	3.3%	-	-
36 h	36.1%	21.5%	13%	7.6%	4.6%	-
48 h	53.2%	32.4%	24.6%	23.8%	18.6%	5.6%
72 h	59.5%	43.2%	36.8%	27.6%	25.2%	10.7%

^1^ Unless otherwise stated, A–E: γ-HPS with MS 0.008, 0.017, 0.053, 0.106 and 0.178, respectively.

## Data Availability

Not applicable.

## Supplementary Materials

The following supporting information can be downloaded at: https://www.mdpi.com/article/10.3390/molecules27072119/s1. Figure S1: TGA and DSC traces as a function of temperature for native and γ-HPS.

## Sample Availability

Samples of the compounds are available from the authors.
