# Supplementary material for: Effect of Molar Substitution on the Properties of γ-Hydroxypropyl Starch
Source: Molecules. 2022 Mar 25;27(7):2119. doi: 10.3390/molecules27072119 (PMC9000244; doi:10.3390/molecules27072119)
Supplement: Supplementary file 1 [file molecules-27-02119-s001.zip › molecules-1623956-supplementary.pdf]

SUPPORTING INFORMATION

Effect of Molar Substitution on the Properties of γ-hydroxypropyl starch

Xue-Li Liu, [ab*] Yi-Fan Chen, [a] Jing-Jing Yang, [a] Si-Jin Li, [a] Hua-Le Xie, [a] Tian-Lin Ma, [a]

[a] *College of Material and Chemical Engineering, Chuzhou University, Anhui 239012, China.*

[b] *School of Chemistry & Chemical Engineering, Anhui University, Hefei 230601, China*

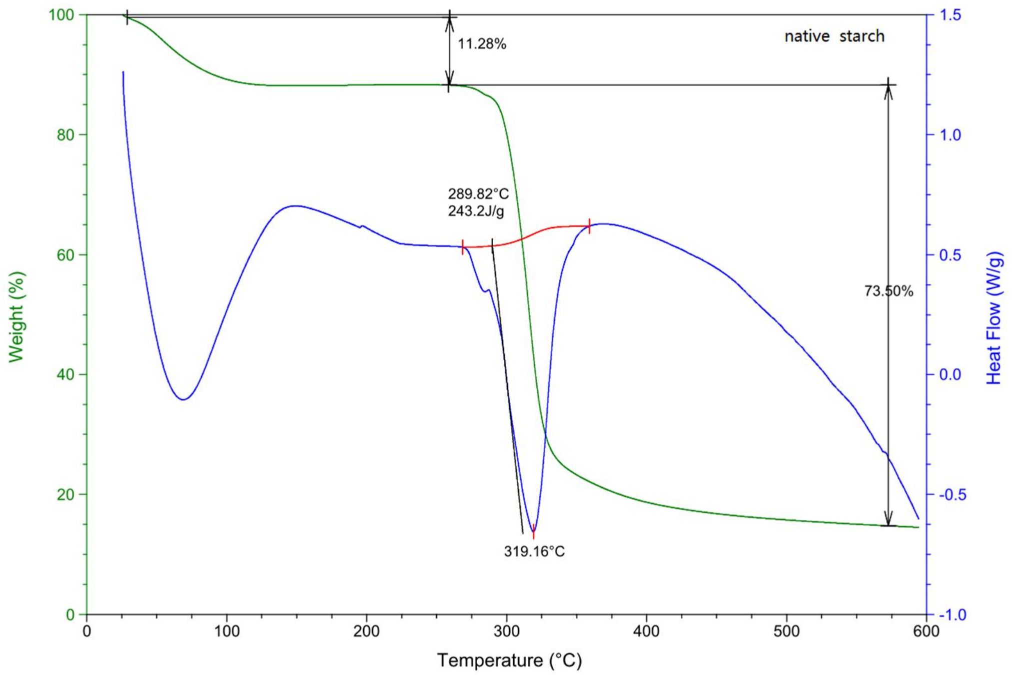
native starch
11.28%
289.82°C
243.2J/g
73.50%
319.16°C
Weight (%)
Heat Flow (W/g)
Temperature (°C)

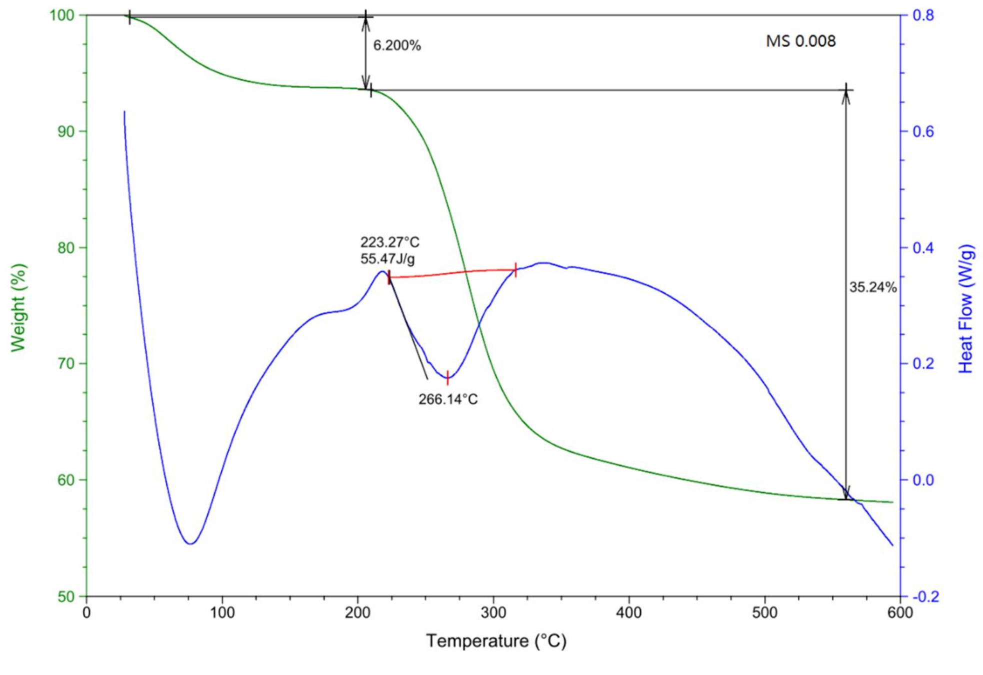
MS 0.008
6.200%
223.27°C
55.47J/g
266.14°C
35.24%
Weight (%)
Heat Flow (W/g)
Temperature (°C)

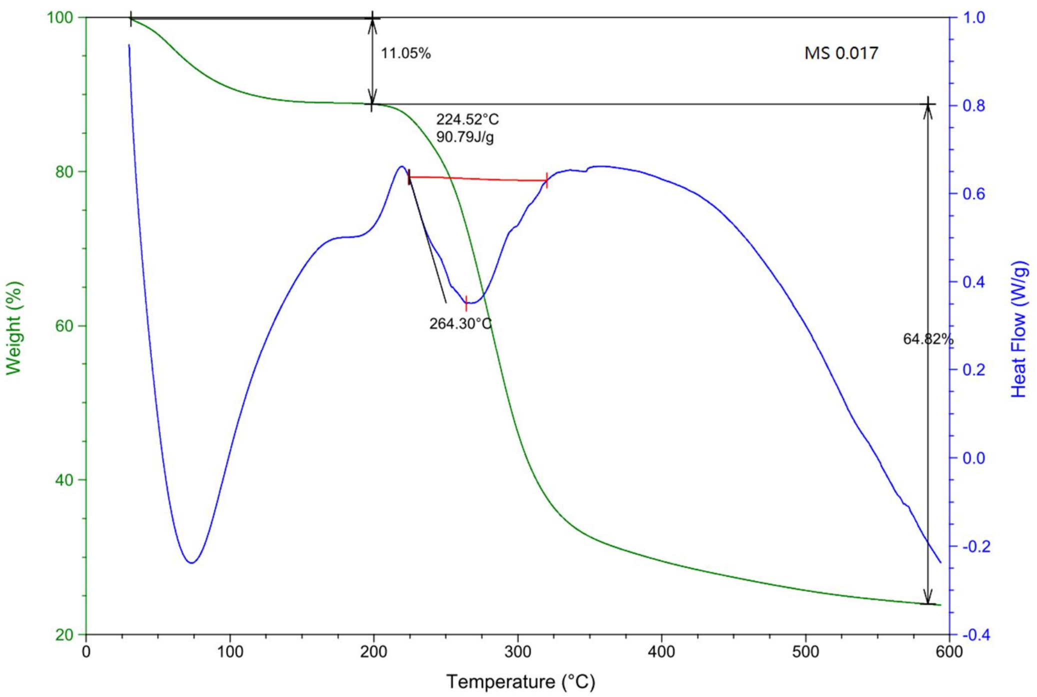
MS 0.017
11.05%
224.52°C
90.79J/g
264.30°C
64.82%
Weight (%)
Heat Flow (W/g)
Temperature (°C)

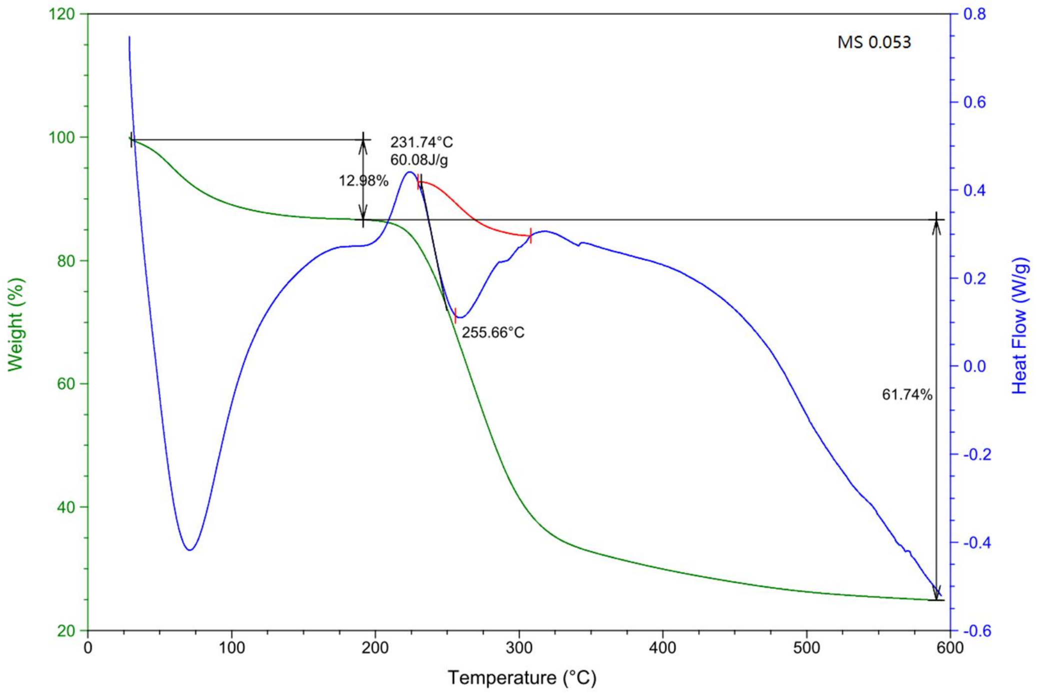
MS 0.053
231.74°C
60.08J/g
12.98%
255.66°C
61.74%
Weight (%)
Heat Flow (W/g)
Temperature (°C)

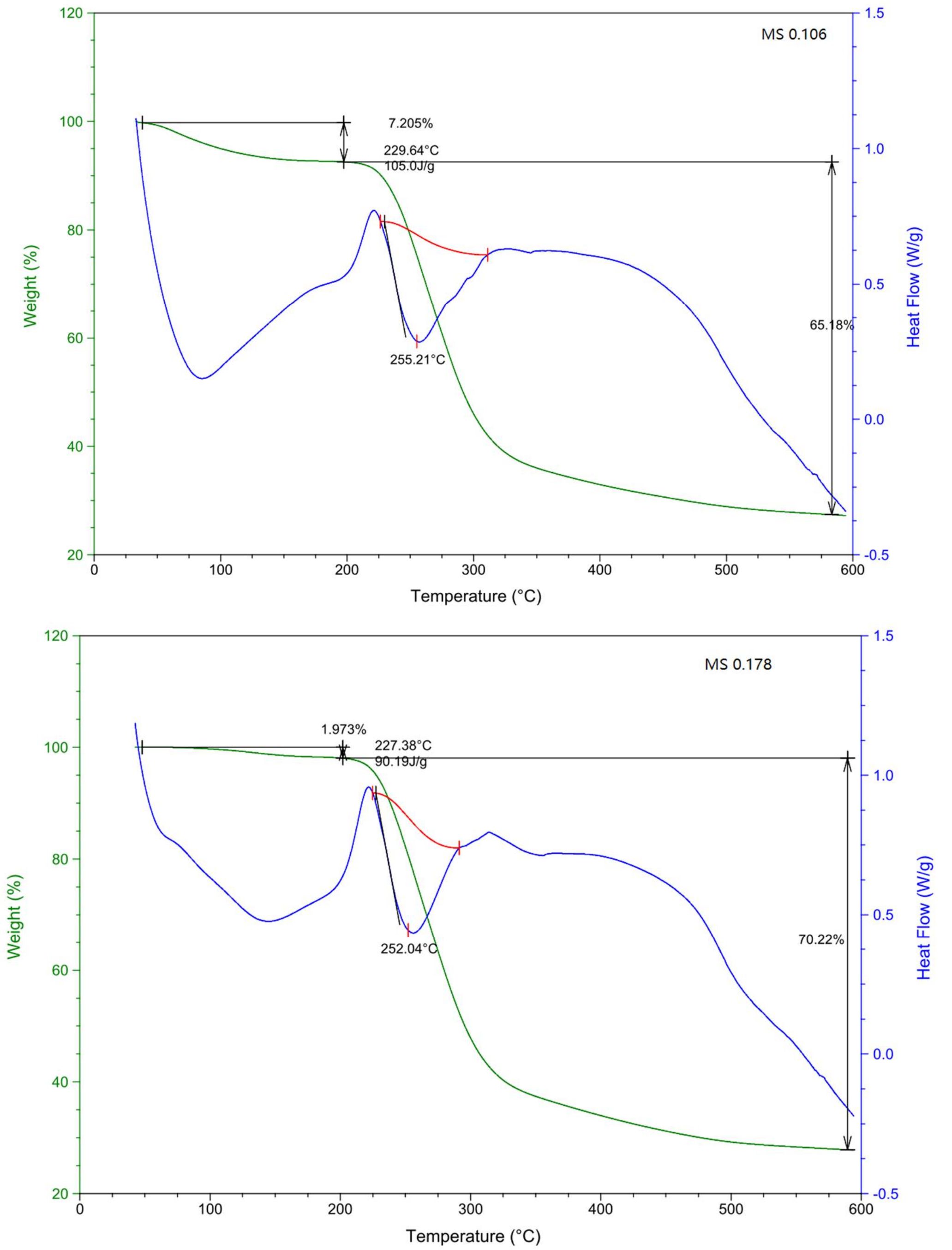

Figure S1. TGA and DSC traces as a function of temperature for native and γ-HPS.
